# Association of vessel density with retinal sensitivity in patients with branch retinal artery occlusion

**DOI:** 10.1371/journal.pone.0328382

**Published:** 2025-07-17

**Authors:** Rei Arasaki, Jacob Yuhang Chin, Tatsuya Inoue, Ryo Asaoka, Kazushi Hirono, Jared Ching, Shin Tanaka, Naoki Soga, Yasuo Yanagi, Maiko Maruyama-Inoue, Kazuaki Kadonosono

**Affiliations:** 1 Department of Ophthalmology and Micro-Technology, Yokohama City University, Kanagawa, Japan; 2 National Healthcare Group, Eye Institute, Tan Tock Seng Hospital, Singapore; 3 Department of Ophthalmology, Seirei Hamamatsu General Hospital, Shizuoka, Japan; 4 Seirei Christopher University, Shizuoka, Japan; 5 Department of Ophthalmology, Addenbrookes Hospital, Cambridge, United Kingdom; Shinshu University School of Medicine, JAPAN

## Abstract

**Purpose:**

To investigate the relationship between vessel density and retinal sensitivity in patients with branch retinal artery occlusion (BRAO).

**Methods:**

This was a retrospective study of fourteen eyes of 13 BRAO patients. MP-3 microperimeter measurement and OCTA exams were performed at the same time. The exams were performed twice, one month and 6 months after the onset. The Early Treatment Diabetic Retinopathy Study (ETDRS) grid was superimposed onto the obtained images. We calculated the retinal sensitivity from MP-3 image, and vessel densities of superficial and deep capillary plexus (sVD and dVD) from OCTA images. These parameters were calculated for both the whole and parafoveal areas. A linear mixed model was used to investigate the correlations between retinal sensitivity and OCTA parameters.

**Results:**

Mean retinal sensitivity was correlated only with sVD for both the whole and parafoveal areas at the first measurement (mR^2^=0.337, p<;0.001 and mR^2^=0.314, p<;0.001, respectively, linear mixed model). A significant correlation was also observed at the second measurement. Moreover, the sVD at the first measurement was significantly associated with retinal sensitivity at the final visit for the whole area (mR^2^=0.364, p<;0.001) and parafoveal areas (mR^2^=0.341, p<;0.001).

**Conclusions:**

The retinal sensitivity of BRAO patients was correlated with the vessel density of superficial capillary plexus at both early and late periods. The superficial vessel density at the first visit was significantly associated with retinal sensitivity at the final visit, implying that early recovery of retinal blood flow is critical for maintaining visual function in RAO eyes.

## Introduction

Branch retinal artery occlusion (BRAO) is known as an ocular vascular occlusive disorder. The most common cause of BRAO is an embolism, while non-embolic causes include vasculitidies such as ocular toxoplasmosis and Behcet’s disease, [1] and vasospasm. In BRAO eyes, visual acuity is relatively preserved as compared to eyes with central retinal artery occlusion (CRAO), and a visual field (VF) defect is usually observed. [2,3] There are some reports describing the natural course of VF defects in CRAO patients [4,5]. The earlier works suggest that VF improvement was associated with the severity of retinal ischemia. However, it is possible that the accuracy of VF measurement might be low because of suboptimal fixation in CRAO eyes due to poor visual acuity. Therefore, it is advantageous to study the time-course change of VF defects in BRAO eyes because of their relatively good fixation stability.

Optical coherence tomography angiography (OCTA) has been widely used for assessing vessel density in several retinal diseases, such as diabetic retinopathy, age-related macular degeneration, and retinal vein occlusion. [6–9]. It has gained popularity due to its convenience and non-invasiveness when compared to dye-based fundus angiography. Yang et al. suggested that the vessel densities of the superficial capillary plexus (SCP) and deep capillary plexus (DCP) were significantly reduced in patients with retinal artery occlusion. [10] However, the relationship between vessel density and retinal sensitivity remains elusive.

Some previous studies have attempted to improve the retinal circulation in CRAO patients by using recombinant tissue plasminogen activator (rTPA) in CRAO patients [11,12]. However, to date, there is no definite criteria for surgical treatment for retinal artery occlusions. In order to assess the efficacy of interventions and determine treatment indications, it is important to investigate the factors influencing the visual outcomes in BRAO eyes. Therefore, the present study aims to investigate the relationship between vessel density and retinal sensitivity using OCTA and microperimetry in BRAO. To evaluate the time course changes of these parameters, we examined them twice during the follow-up period.

## Methods

This retrospective observational study was approved by the institutional review board at Yokohama City University Medical center. The study adhered to the tenets of the declaration of Helsinki. 15 eyes of 14 BRAO patients were included in this study, and informed consent was obtained from all participants. They visited our hospital between July 3, 2020 and October 13, 2021 for the first time and were diagnosed based on clinical examination, funduscopic examination, spectral optical coherence tomography (OCT, Heidelberg Engineering, Heidelberg, Germany), and fluorescein angiography. One eye was excluded from this study because the patient underwent surgery during the follow up period. Thus, 14 eyes of 13 BRAO patients were used for the research. The participants underwent OCTA and VF measurements at the first examination one month after the onset and the second examination six months after the onset. These obtained data were assessed on March 3 2022. We had access to information for identifying individual participants during collection.

### OCTA image processing

OCTA images were obtained with AngioPlex OCTA platform on the spectral-domain CIRRUS HD-OCT 6000 (Carl Zeiss Meditec, Inc., Dublin, CA, USA) twice during the follow-up period, one month and six months after the onset. This device can capture at 100000 A-scans per second and has axial and transverse resolutions of 5 μm and 15 μm, respectively.

We obtained the 3 × 3 mm SCP (from internal limiting membrane to outer boundary of inner plexiform layer) and DCP (from inner boundary of inner nuclear layer to outer boundary of outer plexiform layer plus Henle’s fiber layer) images separately [13]

**Fig 1:**
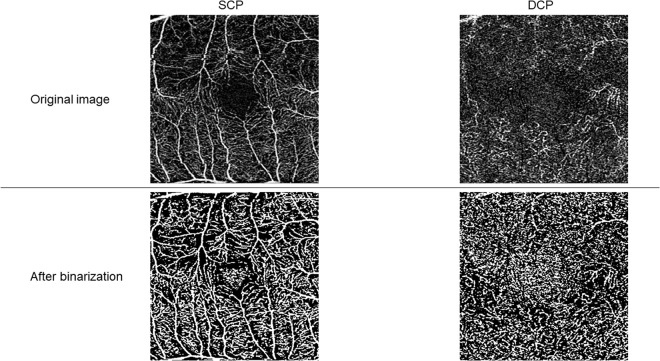
The binarization of OCTA images. The original OCTA images (upper panel) were binarized using Otsu method (lower panel) in the SCP and DCP, respectively. SCP: superficial capillary plexus. DCP: deep capillary plexus.

The OCTA images were imported to ImageJ software (National Institutes of Health [NIH],

Bethesda, MD) and converted into 8-bit format and binarized with Otsu protocol (Fig 1).

**Fig 2:**
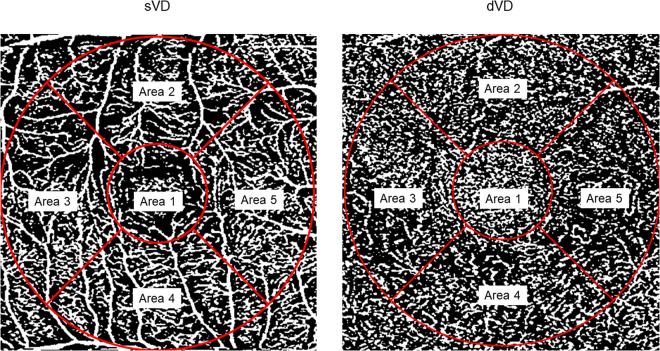
The measurement of vessel density in the total and parafoveal areas. The ETDRS grid was overlaid to the OCTA images (SCP and DCP) and the vessel density was measured for the analysis. We calculated the vessel density within the whole 3-mm circle (t-sVD, t-dVD). We also calculated that of parafoveal areas as the average of Area 2-5 each (sVD, dVD, respectively). t-sVD: vessel density of the superficial layer in the total area. t-dVD: vessel density of the deep layer in the total area. sVD: vessel density of the superficial layer in the parafoveal area. dVD: vessel density of the deep layer in the parafoveal area.

The binarized images were divided into 5 areas with Early Treatment Diabetic Retinopathy Study (ETDRS) grid as shown in previous report [14]. We calculated the vessel density within the whole 3-mm circle (t-sVD, t-dVD) for each eye. Then we also calculated the vessel density of four parafoveal areas for the SCP and DCP (sVD and dVD, Fig 2) and 4 x 14 parafoveal areas were used for statistical analysis.

Simultaneously, we measured the sVD at the first and second examination (sVD1, sVD2) and the dVD at the first and second examination (dVD1, dVD2), respectively.

### VF measurement

**Fig 3:**
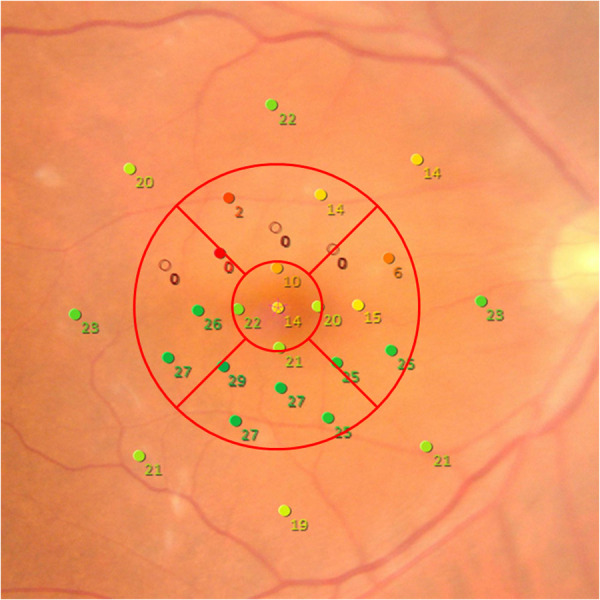
The retinal sensitivity in total and parafoveal areas. The ETDRS grid was overlaid to the MP-3 image. We calculated the mean retinal sensitivity within the whole 3 mm circle (t-MS), and that of parafoveal areas as the average of Area2-5 each (MS) as shown in the previous report [17]. t-MS: mean retinal sensitivity in the total area. MS: mean retinal sensitivity in the parafoveal area.

MP-3 microperimeter (Nidek co.ltd, Aichi, Japan) measurement was performed at the first and second examinations. All patients had a pupil size larger than 4 mm in diameter, as required for the MP-3 measurement. The MP-3 measurement was conducted using the 4–2 full threshold staircase strategy using the standard Goldmann III stimulus size as previously described. [15,16] The ETDRS grid was superimposed onto the MP-3 images and mean sensitivity within the whole 3-mm circle (t-MS) and mean retinal sensitivity of four parafoveal regions (MS) were calculated for each eye (Fig 3).

### Statistical analysis

To compare variables between the first and second examinations, the linear mixed model was used with patient ID as a random effect and timepoint as a fixed effect. Statistical significance was determined based on the p-value associated with the timepoint variable (threshold p < 0.05).

When investigating the associations between retinal sensitivity and each of age and OCTA parameters, univariate linear mixed model was conducted and the marginal R-squared (mR^2^) was calculated to measure the strength of relationship proposed by Nakagawa and Schielzeth. [18]

Then, the associations between t-MS at the first and second examinations (t-MS1, t-MS2) and each of age, t-sVD, t-dVD were investigated using multivariate linear mixed model followed by the corrected Akaike information criterion (AICc) model selection. The AICc, the second-order AIC, provides an accurate estimation even when the sample size is small. [18,19]

Since we obtained the data from a single patient for both eyes, we set the patient ID code as a random effect. Age, sVD and dVD were set as fixed effects. Then, the best model for t-MS1 or t-MS2 was determined from all 2^3^ patterns consisting of three valuables (age, t-sVD, t-dVD).

Regarding the parafoveal region, the following relationships were studied using univariate linear mixed model and multivariate linear mixed model followed by AICc model selection: (i) between MS1 and each of age, sVD1, dVD1, (ii) between MS2 and each of age, sVD2, dVD2, and (iii) between MS2 and each of age, sVD1, dVD1. All statistical analyses were performed using R 4.2.0 (R Foundation for Statistical Computing, Vienna, Austria).

## Results

**Table 1** shows the patients’ demographic data in the current study. The mean age of the patients was 66.3 ± 11.3 years. The logMAR VA of the first and second measurement were −0.022 ± 0.11 and −0.01 ± 0.21, respectively. There was no significant difference in VA between the first and second examinations. (p = 0.70, linear mixed model)

**Table 1:** Demographic data.

	Mean±SD	Range
Age (year)	66.3 ± 11.3	48-80
LogMAR VA1	−0.022 ± 0.11	−0.17-0.3
t-MS1 (dB)	18.5 ± 5.5	9.3-27.4
MS1(dB)	18.6 ± 9.4	0-30.5
t-sVD1 (%)	31.6 ± 4.9	25.8-41.9
sVD1 (%)	32.3 ± 6.4	16.1-45.6
t-dVD1 (%)	30.4 ± 3.6	24.8-39.4
dVD1 (%)	29.4 ± 4.6	11.8-39.7
LogMAR VA2	−0.01 ± 0.21	−0.18-0.70
t-MS2(dB)	18.9 ± 5.4	11.8-27.6
MS2(dB)	19.2 ± 9.1	0-30.25
t-sVD2(%)	29.5 ± 5.4	21.7-39.5
sVD2(%)	30.3 ± 7.4	10.2-41.7
t-dVD2 (%)	27.7 ± 3.9	16.7-32.2
dVD2 (%)	27.2 ± 4.3	12.7-33.4

LogMAR VA1: logarithmic minimum angle of resolution visual acuity at the first examination

t-MS1: mean retinal sensitivity in the total area at the first examination

MS1: mean retinal sensitivity in the parafoveal region at the first examination

t-sVD1: vessel density of the superficial layer in the total area at the first examination

sVD1: vessel density of the superficial layer in the parafoveal area at the first examination

t-dVD1: vessel density of the deep layer in the total area at the first examination

dVD1: vessel density of the deep layer in the parafoveal area at the first examination

LogMAR VA2: logarithmic minimum angle of resolution visual acuity at the second examination

t-MS2: mean retinal sensitivity in the total area at the second examination

MS2: mean retinal sensitivity in the parafoveal region at the second examination

t-sVD2: vessel density of the superficial layer in the total area at the second examination

sVD2: vessel density of the superficial layer in the parafoveal area at the second examination

t-dVD2: vessel density of the deep layer in the total area at the second examination

dVD2: vessel density of the deep layer in the parafoveal area at the second examination

There were no significant changes in t-MS, and t-sVD, and t-dVD between the first and second examinations (p = 0.58, p = 0.12, p = 0.078, respectively, linear mixed model).

At the parafoveal region, there were no significant changes in MS, sVD and dVD between the first and second examinations (p = 0.58, p = 0.12, p = 0.077, respectively, linear mixed model).

At the parafoveal region, there was no significant change in MS between the first and second examinations (p = 0.72), however significant differences were observed in sVD and dVD between the first and second examinations (p = 0.05, p = 0.0039, linear mixed model).

At the first examination, univariate analysis suggested age and t-sVD1 were significantly associated with t-MS1 (mR^2^ = 0.446, p = 0.008 and mR^2^ = 0.337, p < 0.001 respectively, linear mixed model), on the other hand, t-dVD1 was not (p = 0.47, linear mixed model). Multivariate analysis also suggested the optimal model for t-MS1 included only t-sVD1. (Table 2, S1 Fig) Linear mixed model using the parafoveal regions also suggested the sVD1 was correlated with MS1 (Table 3, S2 Fig).

**Table 2:** The optimal model for t-MS1 with linear mixed model.

Variable	Univariate analysis			The optimal model		
fixed-effect coefficient	SE	P value	fixed-effect coefficient	SE	P value
Age	−0.325	0.101	0.008	N.S.		
t-sVD1	0.686	0.000676	<0.001	0.686	0.000676	<0.001
t-dVD1	0.317	0.426	0.47	N.S.		

t-MS1: mean retinal sensitivity in the total area at the first examination

SE: standard error

t-sVD1: vessel density of the superficial layer in the total area at the first examination

t-dVD1: vessel density of the deep layer in the total area at the first examination

**Table 3:** The optimal model for MS1 with linear mixed model.

Variable	Univariate analysis			The optimal model		
	fixed-effect coefficient	SE	P value	fixed-effect coefficient	SE	P value
Age	−0.329	0.103	0.0023	N.S.		
sVD1	0.854	0.181	<0.001	0.854	0.181	<0.001
dVD1	0.409	0.297	0.175	N.S.		

MS1: mean retinal sensitivity in the parafoveal region at the first examination

SE: standard error

sVD1: vessel density of the superficial layer in the parafoveal area at the first examination

dVD1: vessel density of the deep layer in the parafoveal area at the first examination

t-MS1 = −3.21 + 0.686 x t-sVD1 (the random effect description is omitted)

MS1=-9.08 + 0.854 x sVD1 (the random effect description is omitted) (AICc=397.0)

At the second examination, comparable results were obtained. Amongst age, t-sVD2 and t-dVD2, the optimal model for t-MS2 included only t-sVD2. (Table 4, S3 Fig) When analyzing parafoveal regions, amongst age, sVD2 and dVD2, the optimal model for MS2 included only sVD2. (Table 5, S4 Fig)

**Table 4:** The optimal model for t-MS2 with linear mixed model.

Variable	Univariate analysis			The optimal model		
	fixed-effect coefficient	SE	P value	fixed-effect coefficient	SE	P value
Age	−0.265	0.114	0.040	N.S.		
t-sVD2	0.640	<0.001	<0.001	0.640	<0.001	<0.001
t-dVD2	0.854	0.305	0.024	N.S.		

t-MS2: mean retinal sensitivity in the total area at the second examination

SE: standard error

t-sVD2: vessel density of the superficial layer in the total area at the second examination

t-dVD2: vessel density of the deep layer in the total area at the second examination

**Table 5:** The optimal model for MS2 with linear mixed model.

Variable	Univariate analysis			The optimal model		
	fixed-effect coefficient	SE	P value	fixed-effect coefficient	SE	P value
Age	−0.270	0.117	0.040	N.S.		
sVD2	0.913	0.119	<0.001	0.914	0.119	<0.001
dVD2	0.915	0.270	0.002	N.S.		

MS2: mean retinal sensitivity in the parafoveal region at the second examination

SE: standard error

sVD2: vessel density of the superficial layer in the parafoveal area at the second examination

dVD2: vessel density of the deep layer in the parafoveal area at the second examination

Univariate analysis suggested t-sVD1 was correlated with t-MS2 (mR^2^ = 0.364, p < 0.001) but t-dVD1 was not (p = 0.59, linear mixed model). The optimal model for t-MS2 included only t-sVD1 (Table 6, S5 Fig). Furthermore, amongst age, sVD1 and dVD1, the optimal model for MS2 included only sVD1 (Table 7, S6 Fig).

**Table 6:** The optimal model for t-MS2 with linear mixed model.

Variable	Univariate analysis			The optimal model		
	fixed-effect coefficient	SE	P value	fixed-effect coefficient	SE	P value
Age	−0.265	0.114	0.041	N.S.		
t-sVD1	0.744	0.000870	<0.001	0.744	0.000870	<0.001
t-dVD1	0.233	0.420	0.59	N.S.		

t-MS2: mean retinal sensitivity in the total area at the second examination

SE: standard error

t-sVD1: vessel density of the superficial layer in the total area at the first examination

t-dVD1: vessel density of the deep layer in the total area at the first examination

**Table 7:** The optimal model for MS2 with linear mixed model.

Variable	Univariate analysis			The optimal model		
	fixed-effect coefficient	SE	P value	fixed-effect coefficient	SE	P value
Age	−0.271	0.118	0.04	N.S.		
sVD1	0.880	0.175	<0.001	0.880	0.175	<0.001
dVD1	0.248	0.288	0.39	N.S.		

MS2: mean retinal sensitivity in the parafoveal region at the second examination

SE: standard error

sVD1: vessel density of the superficial layer in the parafoveal area at the first examination

dVD1: vessel density of the deep layer in the parafoveal area at the first examination

t-MS2 = −4.40 + 0.744 x t-sVD1 (the random effect description is omitted) (AICc = 91.2)

MS2=-9.22 + 0.880 x sVD1 (the random effect description is omitted) (AICc=391.5)

**Fig 4:**
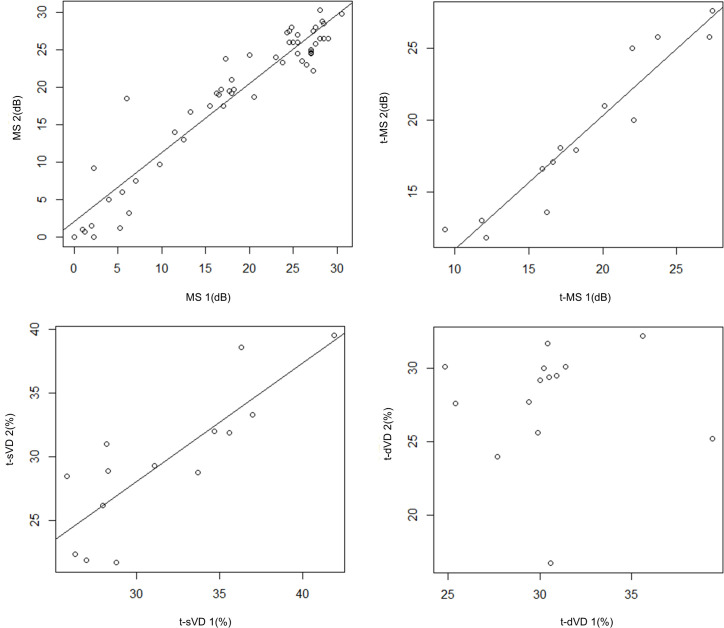
Correlation of MS, sVD and dVD between first and second measurement. MS2 was significantly correlated with MS1 (mR^2^ = 0.902, p < 0.001, linear mixed model), and t-MS2 was correlated with t-MS1 (mR^2^ = 0.919, p < 0.001, linear mixed model). On the other hand, a trend toward significance was observed between t-sVD1 and t-sVD2 (mR^2^ = 0.710, p = 0.09), while t-dVD2 was not correlated with t-dVD1 (p = 0.98, linear mixed model). MS1: mean retinal sensitivity in the parafoveal region at the first examination. MS2: mean retinal sensitivity in the parafoveal region at the second examination. t-MS1: mean retinal sensitivity in the total area at the first examination. t-MS2: mean retinal sensitivity in the total area at the second examination. t-sVD1: vessel density of the superficial layer in the total area at the first examination. t-sVD2: vessel density of the superficial layer in the total area at the second examination. t-dVD1: vessel density of the deep layer in the total area at the first examination. t-dVD2: vessel density of the deep layer in the total area at the second examination.

Univariate analysis suggested MS2 was significantly correlated with MS1 (mR^2^ = 0.902, p < 0.001, linear mixed model) and t-MS2 was correlated with t-MS1 (mR^2^ = 0.919, p < 0.001, linear mixed model). Regarding the vessel density, a trend toward significance was observed between t-sVD1 and t-sVD2 (mR^2^ = 0.710, p = 0.09), however t-dVD2 was not correlated with t-dVD1 (p = 0.98, linear mixed model). (Fig.4)

## Discussion

In this study, we investigated the relationships between the vessel densities and retinal sensitivity in BRAO patients. We found that vessel density of the superficial retina was correlated with retinal sensitivity throughout the follow-up period and the vessel density of SCP at the first examination was correlated to retinal sensitivity at the second examination. In general, visual acuity is relatively maintained in BRAO, therefore retinal sensitivity is one of the most important visual functions to assess. There are a few reports regarding the time course change of the retinal sensitivity in BRAO patients [20,21], but no previous reports investigated the relationship between the perfusion status and retinal sensitivity. As there was a significant correlation between the perfusion status and retinal sensitivity throughout the observation period, it suggests that improving retinal perfusion as early as possible in RAO patients will have a positive effect on retinal sensitivity. Earlier studies report an improvement in the retinal blood flow after administration of rTPA in CRAO eyes [11,12], however the optimal timing for these treatments still remains unclear.

Chen et al. previously reported that intravenous rTPA administration should be performed within 6 hours of onset [22]. On the other hand, in Kadonosono et al’s study, it was proposed that vitrectomy with cannulation of central retinal artery and injection of rTPA with a 47-gauge microneedle may improve the visual outcomes in this group of patients.[12] The study showed that visual acuity significantly improved after treatment, despite having an average interval between the onset and vitrectomy surgery of 37.8 hours (26–47 hours). Our current study suggests that it may be beneficial to improve the retinal blood flow in the early phase in BRAO patients, but the precise timing of intervention remains elusive. Further studies are needed to clarify the timing of intervention in RAO.

Previous OCTA studies have suggested the disruption of superficial and deep capillary plexus in CRAO patients. [23]

There are some reports that show the change in VF defects in CRAO patients. [4,5] Hayreh et al. demonstrated VF defect improvement in 28% of CRAO patients in 30 days follow-up, and Kim et al. showed that 64% of incomplete CRAO patients exhibited VF improvements after at least 6 months’ interval. However, to the best of our knowledge, there are no reports that investigated the relationship between OCTA parameters and VF defects. Furthermore, CRAO patients often had very poor visual acuity and severe fixation loss during OCTA measurement or VF testing. As such, it is possible that the reliability of OCTA imaging and VF tests might be low in CRAO. In contrast, the visual acuity of BRAO patients is relatively good, so the test reliability might be better than CRAO patients. Therefore, the present study is valuable to precisely assess the visual prognosis in RAO.

Despite our careful search of the literature, this is the first study which examines the relationship between OCTA parameters and retinal sensitivity in BRAO patients.

Lu et al. investigated the relationship between the retinal vessel density of OCTA images and visual acuity in RAO patients, and found that superficial vessel density was correlated to visual acuity significantly but deep vessel density was correlated without statical significance.

[24] We used the retinal sensitivity as a visual outcome measure, as it is considered to be a more specific barometer of visual function.

In this study, only the superficial vessel density was correlated with the retinal sensitivity at both first and second measurement.

Yu et al. demonstrated the features of OCT images and their time-series changes with RAO patients previously. Briefly, SCP ischemia corresponded to thickening and hyperreflectivity of the inner retinal layers, but the thickened layers progressed to thinning as the acute RAO areas transitioned into its chronic phase. [25] As the SCP in the OCTA image represents the blood flow of the inner retinal layer [26], it seems reasonable that retinal sensitivity was correspondingly reduced as the superficial retinal blood flow decreased.

In this study, we found that not only sVD but also dVD decreased during 6-month follow-up, whereas there was no difference in retinal sensitivity. It still remains unclear whether vessel density gradually decreases for a long time or not, however further studies are needed to clarify longitudinal changes in vessel density and retinal sensitivity in patients with RAO.

There are some limitations in our study. Firstly, the sample size of this study was relatively small. Secondly, the observation period was relatively short, therefore further studies with larger sample sizes and longer-term follow-up are required. Lastly, there are few reports which investigated the vessel density or retinal sensitivity in eyes with BRAO compared to CRAO. It is possible that our current result doesn’t fit well to CRAO due to the difference in pathology.

In conclusion, we demonstrated significant correlations between the superficial retinal vessel density and the retinal sensitivity at both early and late periods. It was also found that the superficial vessel density at the first visit was significantly associated with retinal sensitivity at the final visit, providing the possibility that early recovery of retinal blood flow might be critical for maintaining visual function in RAO eyes.

## Supporting information

S1 Fig Correlation between t-MS1 and each of age, t-sVD1 and t-dVD1. Age and t-sVD1 were associated with t-MS1 (mR^2^ = 0.446, p = 0.008 and mR^2^ = 0.337, p < 0.001, respectively, linear mixed model), on the other hand, t-dVD1 was not (p = 0.47, linear mixed model). t-MS1: mean retinal sensitivity in the total area at the first examination. t-sVD1: vessel density of the superficial layer in the total area at the first examination. t-dVD1: vessel density of the deep layer in the total area at the first examination.
(TIF)

S2 Fig Correlation between MS1 and each of age, sVD1 and dVD1. Age and sVD1 were correlated with MS1 (mR^2^ = 0.156, p = 0.0023, mR^2^ = 0.314, p < 0.001, respectively, linear mixed model), but dVD1 was not (p = 0.175, linear mixed model). MS1: mean retinal sensitivity in the parafoveal region at the first examination. sVD1: vessel density of the superficial layer in the parafoveal area at the first examination. dVD1: vessel density of the deep layer in the parafoveal area at the first examination.
(TIF)

S3 Fig Correlation between t-MS2 and each of age, t-sVD2 and t-dVD2. Age, t-sVD2, and t-dVD2 were significantly correlated with t-MS2 (mR^2^ = 0.299, p = 0.04, mR^2^ = 0.465, p < 0.001, mR^2^ = 0.334, p = 0.02, respectively, linear mixed model). t-MS2: mean retinal sensitivity in the total area at the second examination. t-sVD2: vessel density of the superficial layer in the total area at the second examination. t-dVD2: vessel density of the deep layer in the total area at the second examination.
(TIF)

S4 Fig Correlation between MS2 and each of age, sVD2 and dVD2. Age, sVD2, and dVD2 were correlated with MS2(mR^2^ = 0.110, p = 0.04, mR^2^ = 0.535, p < 0.001, mR^2^ = 0.186, p = 0.002, respectively, linear mixed model). MS2: mean retinal sensitivity in the parafoveal region at the second examination. sVD2: vessel density of the superficial layer in the parafoveal area at the second examination. dVD2: vessel density of the deep layer in the parafoveal area at the second examination.
(TIF)

S5 Fig Correlation between t-MS2 and each of age, t-sVD1 and t-dVD1. Age, t-sVD1 were correlated with t-MS2 (mR^2^ = 0.299, p = 0.040, mR^2^ = 0.364, p < 0.001, respectively, linear mixed model), but t-dVD1 was not (p = 0.59, linear mixed model). t-MS2: mean retinal sensitivity in the total area at the second examination. t-sVD1: vessel density of the superficial layer in the total area at the first examination. t-dVD1: vessel density of the deep layer in the total area at the first examination.
(TIF)

S6 Fig Correlation between MS2 and each of age, sVD1 and dVD1. Age and sVD1 were correlated with MS2(mR^2^ = 0.110, p = 0.04, mR^2^ = 0.341, p < 0.001, respectively, linear mixed model) but dVD1 was not (p = 0.39, linear mixed model). MS2: mean retinal sensitivity in the parafoveal region at the second examination. sVD1: vessel density of the superficial layer in the parafoveal area at the first examination. dVD1: vessel density of the deep layer in the parafoveal area at the first examination.
(TIF)

S7 Appendix Our minimal data set of this study. (CSV)
