# Supplementary figures and images for: Association of vessel density with retinal sensitivity in patients with branch retinal artery occlusion
Source: PLoS One. 2025 Jul 17;20(7):e0328382. doi: 10.1371/journal.pone.0328382 (PMC12270102; doi:10.1371/journal.pone.0328382)

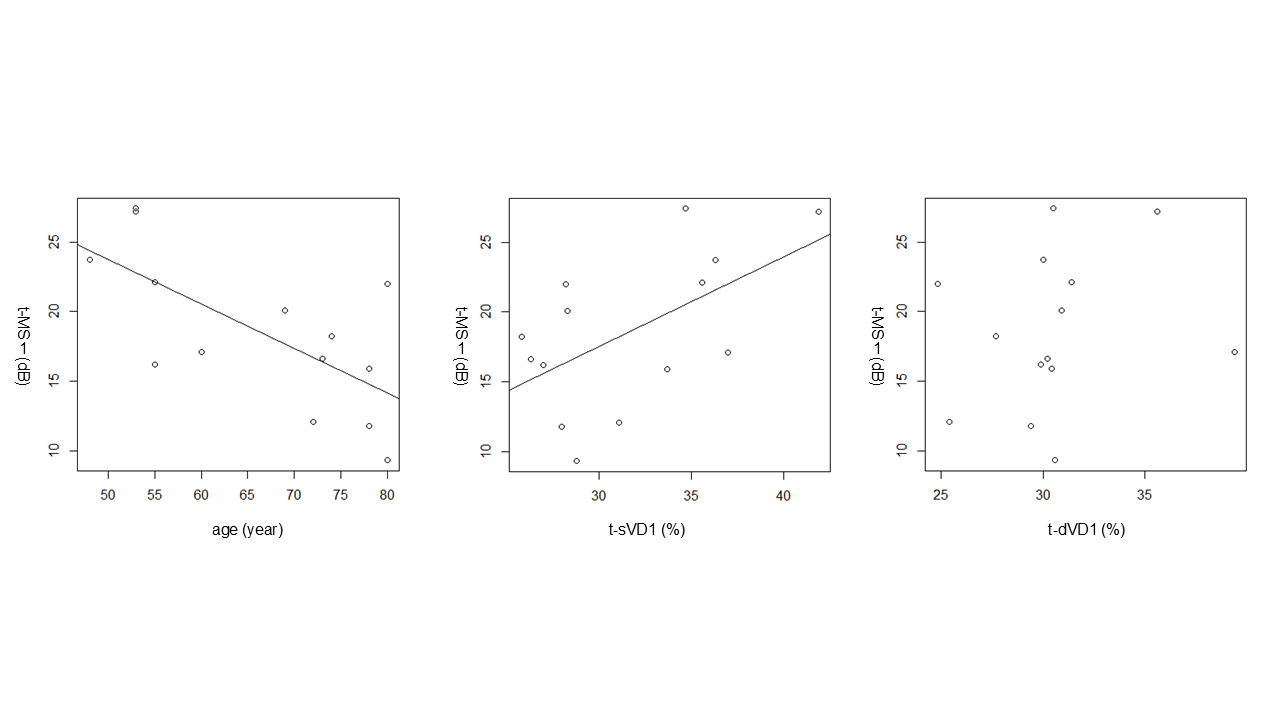

Supplement: S1 Fig — Age and t-sVD1 were associated with t-MS1 (mR2 = 0.446, p = 0.008 and mR2 = 0.337, p < 0.001, respectively, linear mixed model), on the other hand, t-dVD1 was not (p = 0.47, linear mixed model). t-MS1: mean retinal sensitivity in the total area at the first examination. t-sVD1: vessel density of the superficial layer in the total area at the first examination. t-dVD1: vessel density of the deep layer in the total area at the first examination. (TIF) [file pone.0328382.s001.tif]

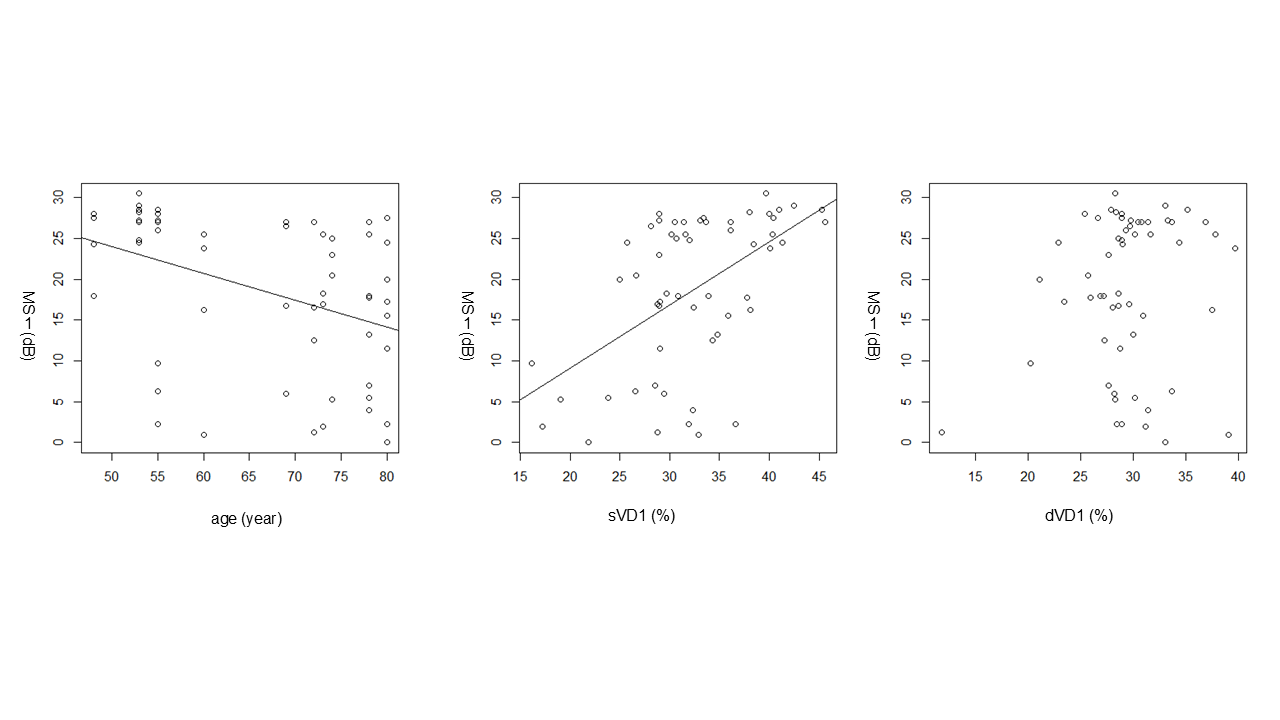

Supplement: S2 Fig — Age and sVD1 were correlated with MS1 (mR2 = 0.156, p = 0.0023, mR2 = 0.314, p < 0.001, respectively, linear mixed model), but dVD1 was not (p = 0.175, linear mixed model). MS1: mean retinal sensitivity in the parafoveal region at the first examination. sVD1: vessel density of the superficial layer in the parafoveal area at the first examination. dVD1: vessel density of the deep layer in the parafoveal area at the first examination. (TIF) [file pone.0328382.s002.tif]

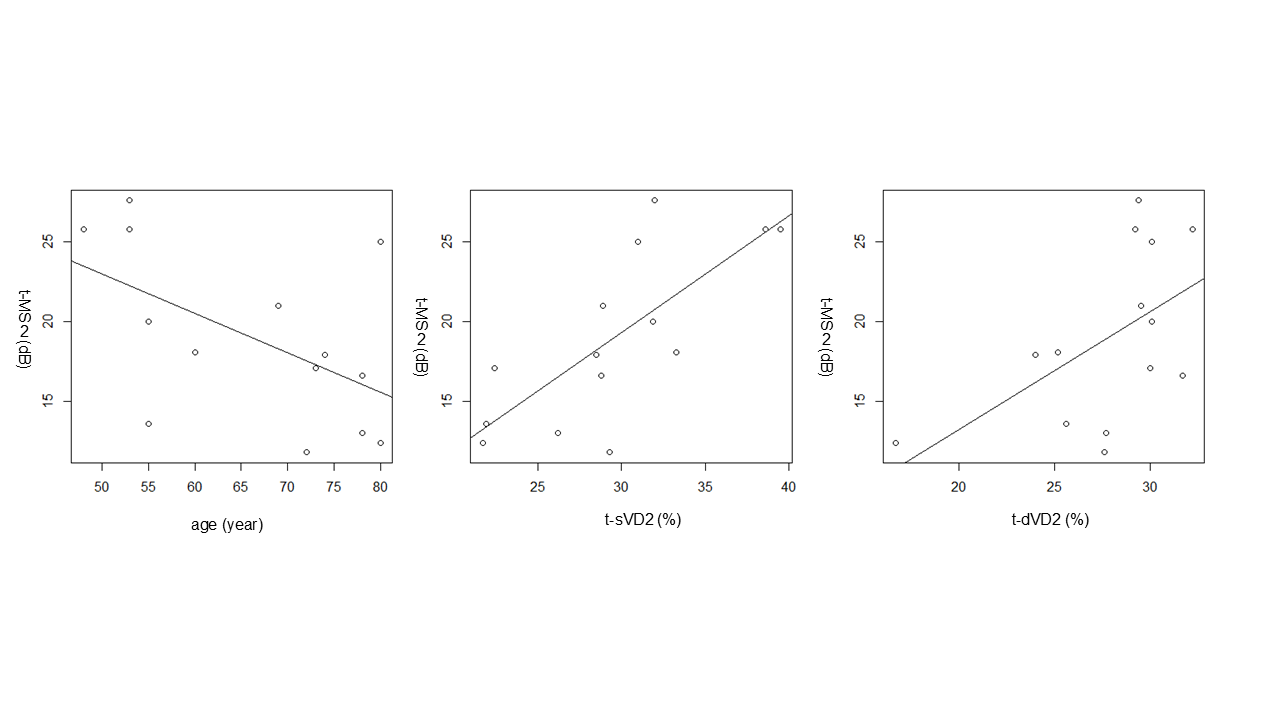

Supplement: S3 Fig — Age, t-sVD2, and t-dVD2 were significantly correlated with t-MS2 (mR2 = 0.299, p = 0.04, mR2 = 0.465, p < 0.001, mR2 = 0.334, p = 0.02, respectively, linear mixed model). t-MS2: mean retinal sensitivity in the total area at the second examination. t-sVD2: vessel density of the superficial layer in the total area at the second examination. t-dVD2: vessel density of the deep layer in the total area at the second examination. (TIF) [file pone.0328382.s003.tif]

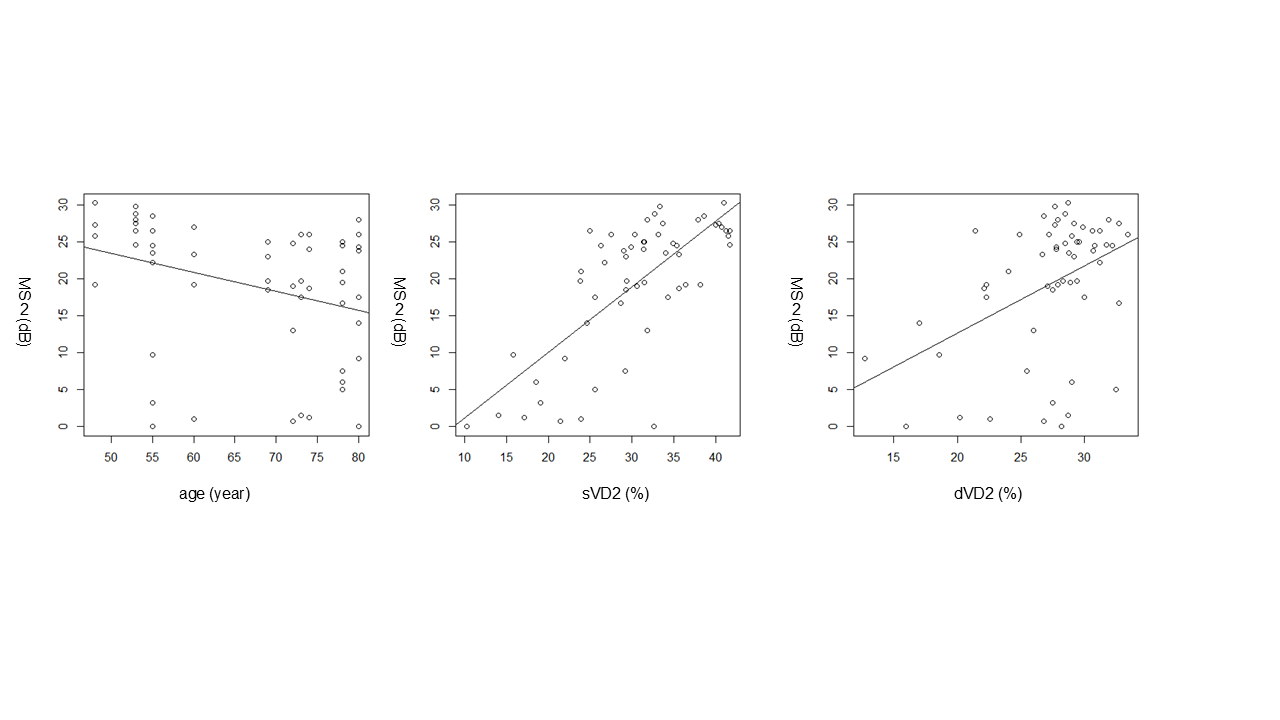

Supplement: S4 Fig — Age, sVD2, and dVD2 were correlated with MS2(mR2 = 0.110, p = 0.04, mR2 = 0.535, p < 0.001, mR2 = 0.186, p = 0.002, respectively, linear mixed model). MS2: mean retinal sensitivity in the parafoveal region at the second examination. sVD2: vessel density of the superficial layer in the parafoveal area at the second examination. dVD2: vessel density of the deep layer in the parafoveal area at the second examination. (TIF) [file pone.0328382.s004.tif]

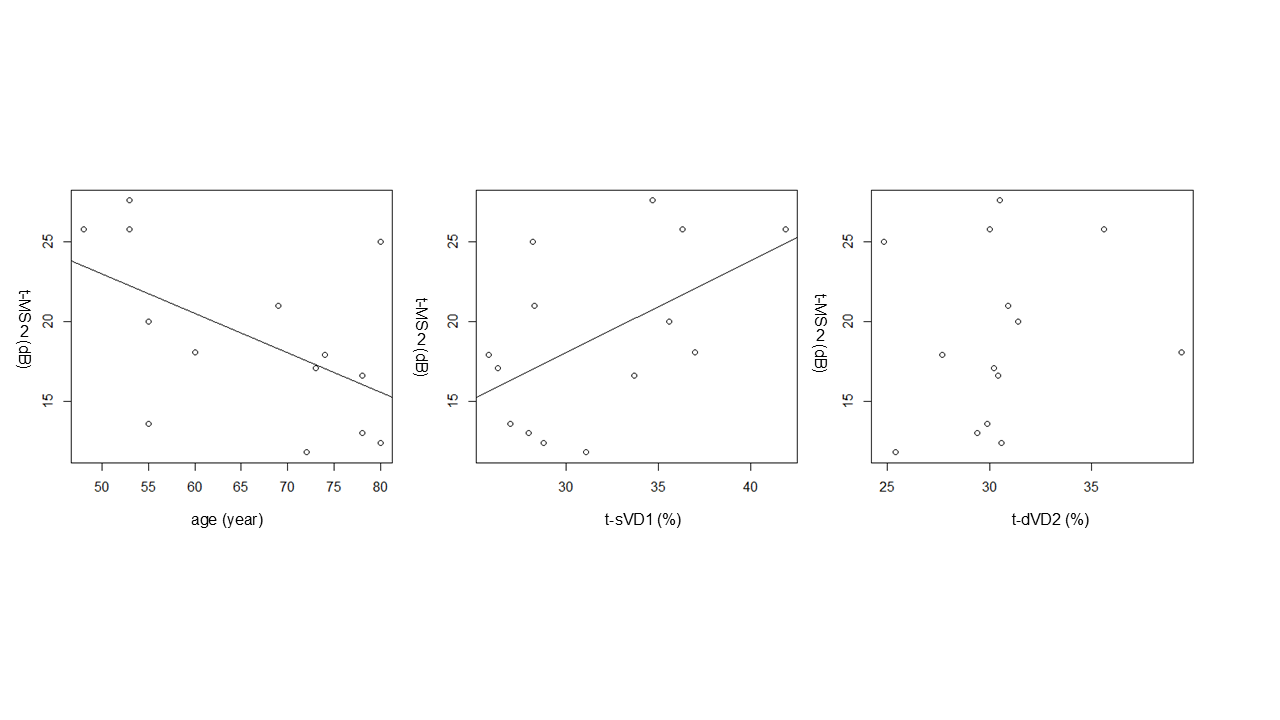

Supplement: S5 Fig — Age, t-sVD1 were correlated with t-MS2 (mR2 = 0.299, p = 0.040, mR2 = 0.364, p < 0.001, respectively, linear mixed model), but t-dVD1 was not (p = 0.59, linear mixed model). t-MS2: mean retinal sensitivity in the total area at the second examination. t-sVD1: vessel density of the superficial layer in the total area at the first examination. t-dVD1: vessel density of the deep layer in the total area at the first examination. (TIF) [file pone.0328382.s005.tif]

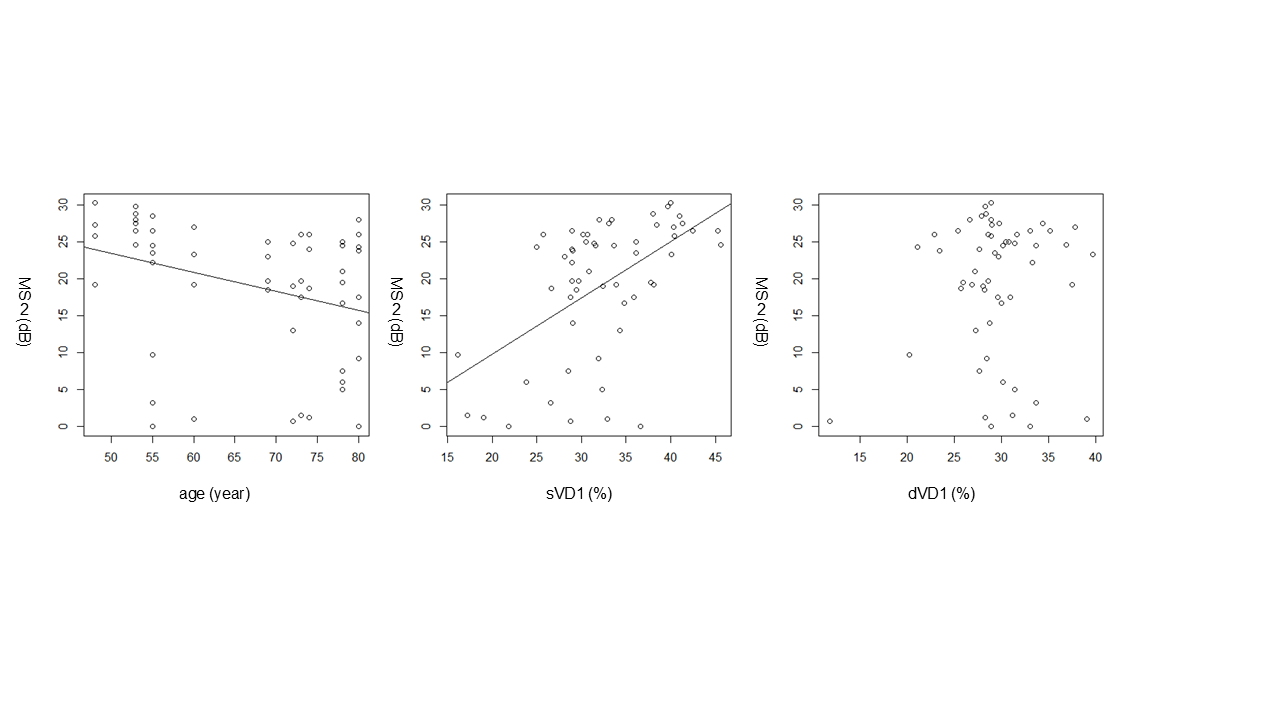

Supplement: S6 Fig — Age and sVD1 were correlated with MS2(mR2 = 0.110, p = 0.04, mR2 = 0.341, p < 0.001, respectively, linear mixed model) but dVD1 was not (p = 0.39, linear mixed model). MS2: mean retinal sensitivity in the parafoveal region at the second examination. sVD1: vessel density of the superficial layer in the parafoveal area at the first examination. dVD1: vessel density of the deep layer in the parafoveal area at the first examination. (TIF) [file pone.0328382.s006.tif]
